# Crystal structure of tetra­kis­[μ-2-(meth­oxy­carbon­yl)benzoato-κ^2^ *O* ^1^:*O* ^1′^]bis­[(*N*,*N*-di­methyl­formamide-κ*O*)copper(II)](*Cu*—*Cu*) di­methyl­formamide disolvate

**DOI:** 10.1107/S2056989018005893

**Published:** 2018-04-24

**Authors:** Jinglin Wang, Feng Su, Lili Shi

**Affiliations:** a Department of Chemistry, Changzhi University, Changzhi, Shanxi 046011, People’s Republic of China

**Keywords:** 2-(meth­oxy­carbon­yl)benzoate, binuclear copper compound, supra­molecular structure, π–π stacking inter­actions, crystal structure

## Abstract

The title compound, [Cu_2_(C_9_H_7_O_4_)_4_(C_3_H_7_NO)_2_]·2C_3_H_7_NO, crystallizes in the monoclinic *P*2_1_/*c* space group, with the binuclear copper unit occupying a special position on an inversion center, *i.e.* the asymmetric unit of the crystal consists of one Cu^II^ ion, two 2-(meth­oxy­carbon­yl)benzoate ligands, and two DMF mol­ecules (one coordinated and one solvate). The binuclear complex displays a paddle-wheel-shaped structure with the two copper atoms being in a Jahn–Teller-distorted octa­hedral coordination environment. Each 2-(meth­oxy­carbon­yl)benzoate substituent acts as a bridging ligand and links two Cu atoms with a Cu—Cu distance of 2.633 (1) Å. The carboxyl­ate groups of the 2-(meth­oxy­carbon­yl)benzoate ligands adopt bidentate *syn*–*syn* bridging modes, with dihedral angles between the carboxyl­ate planes and the aromatic rings of 18.427 (4) and 43.029 (6)°. In the crystal, adjacent DMF mol­ecules coordinated to copper atoms are arranged in a mutual ‘head-to-tail’ manner by offset face-to-face π–π stacking inter­actions, resulting in chains along the *c-*axis direction. The planes of the coordinated DMF mol­ecules are parallel to each other, the distance between them being 3.33 (1) Å. A three-dimensional structure is assembled from the chains by weak C—H⋯O and C—H⋯π inter­molecular inter­actions involving the DMF solvent mol­ecules. One of the methyl ester groups is disordered over two sites with an occupancy ratio of 0.751 (12):0.249 (12).

## Abstract 2

The binuclear Cu^II^ complex displays a paddle-wheel structure. In the crystal, adjacent DMF mol­ecules coordinated to copper atoms are arranged in a mutual ‘head-to-tail’ manner by offset face-to-face π–π stacking inter­actions, resulting in chains along the *c-*axis direction. These are assembled into a three-dimensional structure by further weak C—H⋯O and C—H⋯π inter­actions involving the DMF solvent mol­ecules.

## Chemical context   

Binuclear Cu^II^ compounds have been an attractive target for chemical research because of their wide range of applications in materials chemistry (Kato *et al.*, 1964 ▸; Farraj *et al.*, 2017 ▸), environmental (Pokharel *et al.*, 2014 ▸) and biological chemistry (Ma & Moulton, 2007 ▸). In crystal engineering, the carboxyl­ate ligands are widely used as linkers in the design of binuclear complexes as they exhibit versatile coordination modes for bonding of different metal ions, including monodentate – chelating and monoatomic bridging, as well as bridging modes in *syn*–*anti*, *anti*–*anti* and *syn*–*syn* conformations (Su *et al.*, 2015 ▸). Thus, carboxyl­ate ligands can adopt μ_2_-*O*, chelate or bridging modes to construct binuclear copper complexes. In addition, the Cu—Cu dimer can be tetra bridged by four carboxyl­ate groups to form a paddle-wheel building unit. Furthermore, the paddle-wheel building unit may be axially coordinated by means of two monodentate ligands to give the formula [Cu_2_(OOC*R*)_4_ *L*_2_] (Suh *et al.*, 2012 ▸). For example, [Cu_2_(aspirinate)_4_ *L*_2_] [*L* = *N*,*N*-di­methyl­formamide (DMF), 3-bromo­pyridine, quinoline, pyridine; Ma & Moulton, 2007 ▸], [Cu_2_(Sal)_4_(aceto­nitrile)_2_] (Sal = salicylate; Liu *et al.*, 2017 ▸), [Cu_2_[2-(meth­oxy­carbon­yl)benzoate]_4_(MeOH)(DMF)], (Liu *et al.*, 2008 ▸), [Cu_2_(2-(meth­oxy­carbon­yl) benzoate)_4_(aceto­nitrile)_2_] (Wang *et al.*, 2013 ▸). In a similar way, binuclear copper coordination polymers (CPs) with paddle-wheel cluster units can be coordinated by functional ligands in the axial position, including 4,4′-bi­pyridine (Liu *et al.*, 2005 ▸), pyrazine (Kitao *et al.*, 2017 ▸), 2,5-bis­(4-pyrid­yl)-1,3,4-oxa­diazole (Hou *et al.*, 2004 ▸), forming a class of multifunctional polymer materials. Moreover, it is well known that the solubility and lipophilicity are the key parameters of drugs, and the appropriate choice of an axial ligand affords the ability to significantly alter these properties.
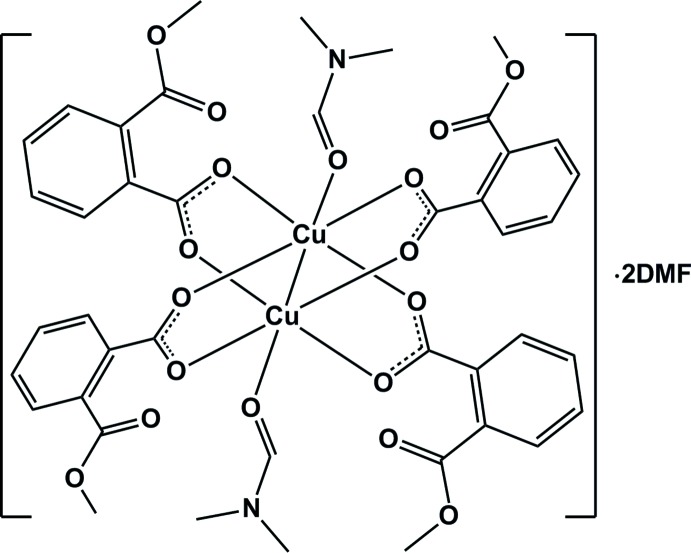


In this paper, we report the synthesis and crystal structure of a new binuclear copper complex [Cu_2_(2-(meth­oxy­carbon­yl)benzoate)_4_(DMF)_2_], (I), containing the paddle-wheel building unit.

## Structural commentary   

**Figure 1:**
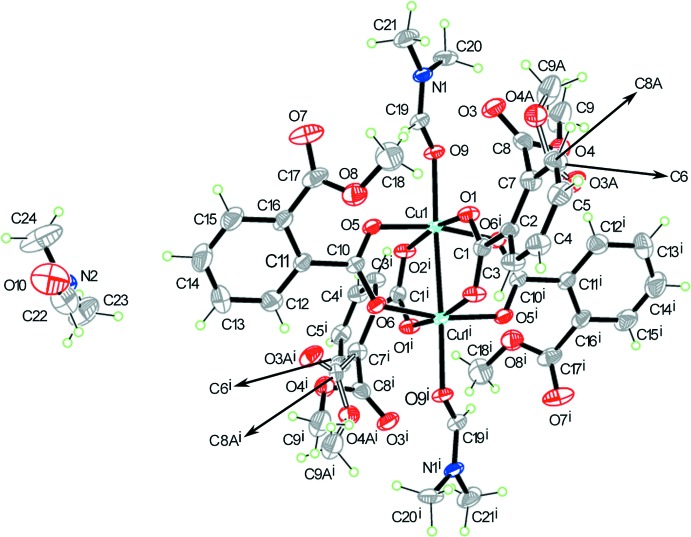
Mol­ecular structure of (I) drawn with 30% probability displacement ellipsoids. Symmetry code: (i) –*x* + 1, –*y* + 1, –*z* + 1.

The title compound crystallizes in the monoclinic *P*2_1_/*c* space group, with the binuclear copper unit occupying a special position on the inversion center. The asymmetric unit consists of one Cu^II^ ion, two 2-(meth­oxy­carbon­yl)benzoate ligands, and two DMF mol­ecules (one coordinated and one solvate). The complex displays a paddle-wheel-shaped binuclear structure (Fig. 1 ▸). If the Cu—Cu bonding contact is neglected, each Cu^II^ ion is penta­coordinated to four carboxyl­ate oxygen atoms [O1, O2^i^, O5 and O6^i^] of four 2-(meth­oxy­carbon­yl)benzoate ligands and one oxygen atom [O9] from the DMF mol­ecule. Both Cu^II^ ions exhibit Jahn–Teller square-pyramidal geometries (*τ* = 0), with four short Cu—O(carboxyl­ate) [1.934 (4) to 1.968 (4) Å; Table 1 ▸] bond lengths in the equatorial plane and one long Cu—O(DMF) [2.132 (4) Å] bond length at the axial position. Each 2-(meth­oxy­carbon­yl)benzoate substituent acts as a bridging ligand and links two Cu atoms with a Cu—Cu(−*x* + 1, −*y* + 1, −*z* + 1) distance of 2.633 (1) Å; this is close to the 2.64 Å reported for the similar dinuclear complex [Cu_2_(OAc)_4_·2H_2_O] (Kato *et al.*, 1964 ▸). However, the Cu—Cu^i^ distance in (I) is slightly longer than the Cu—Cu separation of 2.56 Å in metallic copper (Jones *et al.*, 1997 ▸). The carboxyl­ate groups of the 2-(meth­oxy­carbon­yl)benzoate ligands adopt bidentate *syn–syn* bridging modes, with the dihedral angles between the carboxyl­ate planes and the aromatic rings of 18.427 (4) and 43.029 (6)°.

## Supra­molecular features   

**Figure 2:**
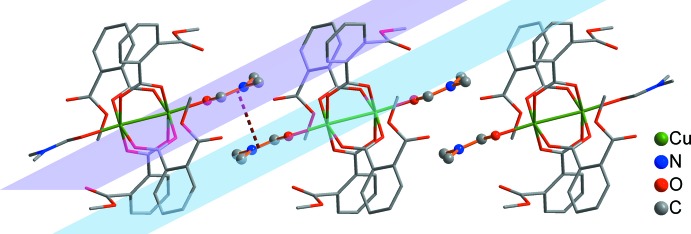
The one-dimensional motif from the binuclear copper fragments of (I) along the *c-*axis direction formed by π–π stacking inter­actions (dashed lines) between the coordinated DMF mol­ecules.

**Figure 3:**
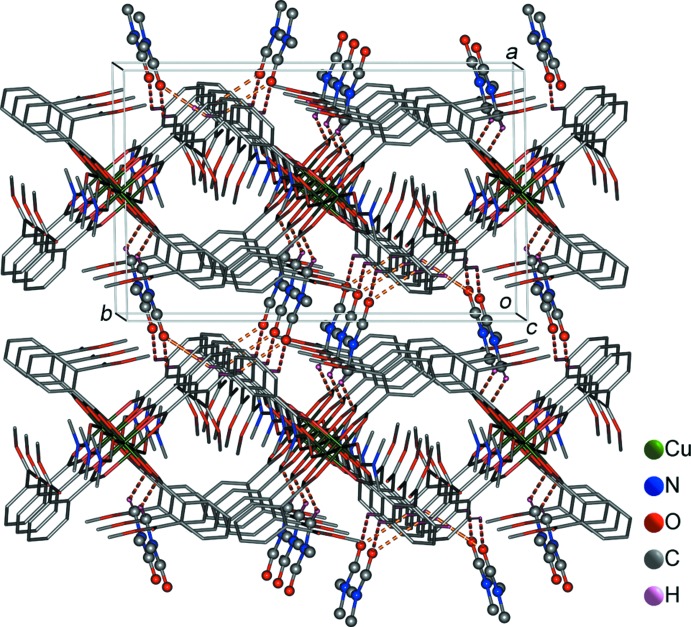
The crystal packing of (I) showing the three-dimensional supra­molecular network along the *c* axis. The inter­molecular C—H⋯O hydrogen bonds are shown as yellow dotted lines.

The crystal structure of (I) contains both coordinated and solvate DMF mol­ecules. As illustrated in Fig. 2 ▸, adjacent DMF mol­ecules coordinated to copper atoms are arranged in a mutual ‘head-to-tail’ manner by offset face-to-face π–π stacking inter­actions (Wang *et al.*, 2010 ▸), resulting in chains along the *c-*axis direction. The planes of the coordinated DMF mol­ecules are parallel to each other, the distance between them being 3.33 (1) Å. The three-dimensional structure of (I) is assembled from these chains by further weak C—H⋯O inter­actions (H⋯*A* distances of 2.63–2.70 Å; Table 2 ▸) and inter­molecular π⋯π inter­actions (Fig. 3 ▸).

## Database survey   

There are a number of Cu paddle-wheel structures [Cu_2_(OOC*R*)_4_ *L*_2_] in the crystallographic literature with benzene carboxyl­ates derivatives (Cambridge Structural Database, Version 5.39, updated in November 2017; Groom *et al.*, 2016 ▸). In most cases, both copper centers in these complexes feature a coordinated water mol­ecule in the axial position, which can be replaced by small solvent mol­ecules to generate potential binding sites; for example, *L* = Cl^−^ (Silva *et al.*, 2001 ▸), urea, ethanol, benzoic acid (Kato *et al.*, 1964 ▸), *N*,*N*-di­methyl­formamide, 3-bromo­pyridine, quinoline, pyridine, isonicotinamide, nicotinamide, 3-phenyl­pyridine (Ma & Moulton, 2007 ▸), aceto­nitrile (Liu *et al.*, 2017 ▸, Wang *et al.*, 2013 ▸), methanol (Liu *et al.*, 2008 ▸), 2-picoline (Del Sesto *et al.*, 2000 ▸). Various polycarb­oxy­lic benzene derivatives have been synthesized to obtain porous coordination polymers (Guillerm *et al.*, 2014 ▸), which exhibit different properties owing to different substituent groups in the axial sites.

## Synthesis and crystallization   

The title complex was synthesized according to a literature procedure (Wang *et al.*, 2013 ▸). 2-(Meth­oxy­carbon­yl)benzoic acid (180.0 mg, 1.0 mmol) and NaOH (40.0 mg, 1.0 mmol) were dissolved in a methanol solution (20 mL) while stirring at room temperature for 20 min. Then, 15 mL of a methanol solution containing Cu(NO_3_)_2_·3H_2_O (121 mg, 0.5 mmol) was added to the mixture, and the mixture was further stirred at room temperature for 90 min. The blue precipitate obtained was separated by filtration, washed with methanol and dried. The powder was dissolved in *N*,*N*-di­methyl­formamide, and blue single crystals were collected after slow evaporation at room temperature for several weeks.

## Refinement   

Crystal data, data collection and structure refinement details are summarized in Table 3 ▸. All hydrogen atoms were positioned geometrically with C—H = 0.93–0.96 Å and refined using the riding model with fixed displacement parameters [*U*_iso_(H) = 1.5*U*_eq_(C) for methyl groups and 1.2*U*_eq_(C) for the other groups]. One of the methyl ester groups is disordered over two sets of sties with an occupancy ratio of 0.751 (12):0.249 (12). The displacement parameters of the O4/O4*A* and C9/C9*A* atoms of the disordered fragment were restrained to be similar (Sheldrick, 2015 ▸).

## Supplementary Material

Crystal structure: contains datablock(s) I. DOI: 10.1107/S2056989018005893/kq2019sup1.cif

Structure factors: contains datablock(s) I. DOI: 10.1107/S2056989018005893/kq2019Isup2.hkl

CCDC reference: 1837401

Additional supporting information:  crystallographic information; 3D view; checkCIF report

## Figures and Tables

**Table 1:** Selected geometric parameters (Å, °)

Cu1—Cu1^i^	2.6329 (14)	Cu1—O5	1.968 (4)
Cu1—O1	1.943 (4)	Cu1—O6^i^	1.953 (4)
Cu1—O2^i^	1.934 (4)	Cu1—O9	2.132 (4)
			
O2^i^—Cu1—O1	168.03 (18)	O6^i^—Cu1—O5	168.06 (18)
O2^i^—Cu1—O6^i^	90.25 (19)	O2^i^—Cu1—O9	99.14 (18)
O1—Cu1—O6^i^	89.05 (19)	O1—Cu1—O9	92.82 (18)
O2^i^—Cu1—O5	88.7 (2)	O6^i^—Cu1—O9	96.36 (17)
O1—Cu1—O5	89.48 (19)	O5—Cu1—O9	95.55 (17)

Symmetry code: (i) 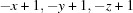.

**Table 2:** Hydrogen-bond geometry (Å, °)

*D*—H⋯*A*	*D*—H	H⋯*A*	*D*⋯*A*	*D*—H⋯*A*
C12—H12⋯O3*A* ^i^	0.93	2.50	3.29 (3)	143
C23—H23*A*⋯O6^ii^	0.96	2.70	3.538 (11)	145
C3—H3⋯O10^iii^	0.93	2.63	3.284 (10)	128
C6—H6⋯O10^iv^	0.93	2.69	3.355 (11)	129

Symmetry codes: (i) 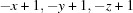; (ii) 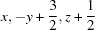; (iii) 
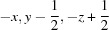
; (iv) 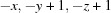.

**Table 3:** Experimental details

Crystal data	
Chemical formula	[Cu_2_(C_9_H_7_O_4_)_4_(C_3_H_7_NO)_2_]·2C_3_H_7_NO
*M*_r_	1136.07
Crystal system, space group	Monoclinic, *P*2_1_/*c*
Temperature (K)	296
*a*, *b*, *c* (Å)	12.7775 (12), 19.746 (2), 10.7957 (11)
β (°)	106.870 (2)
*V* (Å^3^)	2606.6 (4)
*Z*	2
Radiation type	Mo *K*α
μ (mm^−1^)	0.90
Crystal size (mm)	0.45 × 0.34 × 0.21

Data collection	
Diffractometer	Bruker Photon 100
Absorption correction	Multi-scan (*SADABS*; Bruker, 2015 ▸)
*T*_min_, *T*_max_	0.689, 0.834
No. of measured, independent and observed [*I* > 2σ(*I*)] reflections	12822, 4610, 2522
*R*_int_	0.101
(sin θ/λ)_max_ (Å^−1^)	0.596

Refinement	
*R*[*F* ^2^ > 2σ(*F* ^2^)], *wR*(*F* ^2^), *S*	0.061, 0.221, 1.01
No. of reflections	4610
No. of parameters	373
No. of restraints	12
H-atom treatment	H-atom parameters constrained
Δρ_max_, Δρ_min_ (e Å^−3^)	0.91, −0.54

Computer programs: *APEX3* and *SAINT* (Bruker, 2015 ▸), *SHELXS97* and *SHELXTL* (Sheldrick, 2008 ▸), *SHELXL2016* (Sheldrick, 2015 ▸), *DIAMOND* (Brandenburg, 2006 ▸), *PLATON* (Spek, 2009 ▸) and *publCIF* (Westrip, 2010 ▸).

## supplementary crystallographic information

### Crystal data

**Table:**

[Cu_2_(C_9_H_7_O_4_)_4_(C_3_H_7_NO)_2_]·2C_3_H_7_NO	*F*(000) = 1180
*M**_r_* = 1136.07	*D*_x_ = 1.447 Mg m^−^^3^
Monoclinic, *P*2_1_/*c*	Mo *K*α radiation, λ = 0.71073 Å
*a* = 12.7775 (12) Å	Cell parameters from 2608 reflections
*b* = 19.746 (2) Å	θ = 2.2–21.7°
*c* = 10.7957 (11) Å	µ = 0.90 mm^−^^1^
β = 106.870 (2)°	*T* = 296 K
*V* = 2606.6 (4) Å^3^	Block, blue
*Z* = 2	0.45 × 0.34 × 0.21 mm

### Data collection

**Table:**

Bruker Photon 100 diffractometer	2522 reflections with *I* > 2σ(*I*)
Radiation source: sealed tube	*R*_int_ = 0.101
φ and ω scans	θ_max_ = 25.1°, θ_min_ = 1.7°
Absorption correction: multi-scan (*SADABS*; Bruker, 2015)	*h* = −12→15
*T*_min_ = 0.689, *T*_max_ = 0.834	*k* = −23→17
12822 measured reflections	*l* = −12→12
4610 independent reflections	

### Refinement

**Table:**

Refinement on *F*^2^	12 restraints
Least-squares matrix: full	Hydrogen site location: inferred from neighbouring sites
*R*[*F*^2^ > 2σ(*F*^2^)] = 0.061	H-atom parameters constrained
*wR*(*F*^2^) = 0.221	*w* = 1/[σ^2^(*F*_o_^2^) + (0.1098*P*)^2^ + 2.5593*P*] where *P* = (*F*_o_^2^ + 2*F*_c_^2^)/3
*S* = 1.01	(Δ/σ)_max_ < 0.001
4610 reflections	Δρ_max_ = 0.91 e Å^−^^3^
373 parameters	Δρ_min_ = −0.54 e Å^−^^3^

### Special details

**Table:**

Geometry. All esds (except the esd in the dihedral angle between two l.s. planes) are estimated using the full covariance matrix. The cell esds are taken into account individually in the estimation of esds in distances, angles and torsion angles; correlations between esds in cell parameters are only used when they are defined by crystal symmetry. An approximate (isotropic) treatment of cell esds is used for estimating esds involving l.s. planes.

### Fractional atomic coordinates and isotropic or equivalent isotropic displacement parameters (Å^2^)

**Table:**

	*x*	*y*	*z*	*U*_iso_*/*U*_eq_	Occ. (<1)
Cu1	0.51748 (6)	0.48436 (4)	0.62288 (7)	0.0317 (3)	
N1	0.4576 (5)	0.4167 (3)	0.9654 (5)	0.0451 (14)	
N2	0.0957 (5)	0.9334 (4)	0.6092 (6)	0.0641 (19)	
O1	0.4075 (4)	0.4160 (2)	0.5502 (4)	0.0439 (12)	
O2	0.3789 (3)	0.4416 (2)	0.3420 (4)	0.0447 (12)	
O5	0.4004 (3)	0.5502 (2)	0.6162 (4)	0.0413 (11)	
O6	0.3716 (3)	0.5759 (2)	0.4083 (4)	0.0431 (12)	
O7	0.1773 (7)	0.5501 (4)	0.7540 (7)	0.116 (3)	
O8	0.1653 (4)	0.5121 (3)	0.5590 (6)	0.0668 (16)	
O9	0.5355 (3)	0.4523 (2)	0.8165 (4)	0.0409 (11)	
O10	−0.0745 (6)	0.8955 (4)	0.5178 (7)	0.110 (3)	
C1	0.3623 (5)	0.4065 (3)	0.4326 (7)	0.0363 (16)	
C2	0.2811 (5)	0.3508 (3)	0.3972 (6)	0.0341 (15)	
C3	0.2052 (5)	0.3496 (4)	0.2792 (7)	0.0472 (18)	
H3	0.206712	0.382714	0.218399	0.057*	
C4	0.1263 (6)	0.3001 (4)	0.2487 (7)	0.053 (2)	
H4	0.074745	0.300086	0.167725	0.064*	
C5	0.1230 (6)	0.2512 (4)	0.3359 (8)	0.056 (2)	
H5	0.069538	0.217744	0.314463	0.067*	
C6	0.1986 (6)	0.2512 (4)	0.4554 (8)	0.0530 (19)	
H6	0.196603	0.217854	0.515469	0.064*	
C7	0.2772 (6)	0.3005 (4)	0.4863 (7)	0.0476 (18)	
C8	0.3466 (11)	0.3011 (6)	0.6261 (14)	0.048 (3)	0.751 (12)
O3	0.3230 (6)	0.3196 (4)	0.7184 (7)	0.064 (3)	0.751 (12)
O4	0.4429 (10)	0.2714 (6)	0.6315 (11)	0.060 (3)	0.751 (12)
C9	0.5209 (13)	0.2647 (9)	0.7578 (13)	0.097 (6)	0.751 (12)
H9A	0.535773	0.308469	0.797628	0.145*	0.751 (12)
H9B	0.587480	0.245487	0.749156	0.145*	0.751 (12)
H9C	0.491382	0.235598	0.810555	0.145*	0.751 (12)
C8A	0.391 (4)	0.283 (2)	0.580 (5)	0.052 (12)	0.249 (12)
O3A	0.4754 (18)	0.2744 (13)	0.561 (3)	0.064 (8)	0.249 (12)
O4A	0.370 (2)	0.2762 (16)	0.688 (3)	0.060 (4)	0.249 (12)
C9A	0.470 (4)	0.259 (3)	0.798 (5)	0.096 (6)	0.249 (12)
H9A1	0.480823	0.211297	0.802431	0.144*	0.249 (12)
H9A2	0.459759	0.275219	0.877950	0.144*	0.249 (12)
H9A3	0.532924	0.281117	0.784324	0.144*	0.249 (12)
C10	0.3532 (5)	0.5818 (3)	0.5149 (6)	0.0356 (15)	
C11	0.2753 (5)	0.6360 (4)	0.5253 (7)	0.0441 (17)	
C12	0.2796 (7)	0.6975 (4)	0.4697 (8)	0.064 (2)	
H12	0.328128	0.704014	0.421111	0.077*	
C13	0.2133 (8)	0.7503 (5)	0.4842 (10)	0.085 (3)	
H13	0.217287	0.792384	0.447102	0.102*	
C14	0.1418 (9)	0.7390 (6)	0.5547 (11)	0.094 (3)	
H14	0.095559	0.773774	0.564212	0.113*	
C15	0.1368 (7)	0.6792 (6)	0.6101 (9)	0.079 (3)	
H15	0.088721	0.673494	0.659549	0.095*	
C16	0.2008 (6)	0.6260 (4)	0.5959 (7)	0.052 (2)	
C17	0.1818 (6)	0.5593 (5)	0.6481 (9)	0.064 (2)	
C18	0.1415 (8)	0.4457 (5)	0.5937 (11)	0.095 (3)	
H18A	0.131302	0.416073	0.520669	0.143*	
H18B	0.201215	0.429533	0.663889	0.143*	
H18C	0.076007	0.446603	0.620192	0.143*	
C19	0.4544 (6)	0.4433 (4)	0.8545 (6)	0.0433 (17)	
H19	0.386642	0.456500	0.800306	0.052*	
C20	0.5587 (7)	0.3926 (4)	1.0530 (8)	0.072 (3)	
H20A	0.544984	0.374755	1.129558	0.107*	
H20B	0.588580	0.357686	1.011590	0.107*	
H20C	0.609754	0.429424	1.076264	0.107*	
C21	0.3581 (7)	0.4049 (5)	1.0006 (9)	0.078 (3)	
H21A	0.375788	0.385056	1.085330	0.117*	
H21B	0.320836	0.447112	1.000652	0.117*	
H21C	0.311570	0.374657	0.939037	0.117*	
C22	0.0126 (9)	0.9123 (5)	0.5152 (10)	0.089 (3)	
H22	0.024510	0.910232	0.434322	0.107*	
C23	0.1986 (7)	0.9484 (6)	0.5900 (10)	0.105 (4)	
H23A	0.248667	0.963075	0.670171	0.157*	
H23B	0.226912	0.908478	0.560193	0.157*	
H23C	0.189853	0.983615	0.526528	0.157*	
C24	0.0870 (9)	0.9415 (9)	0.7348 (11)	0.149 (6)	
H24A	0.155395	0.957274	0.790841	0.223*	
H24B	0.030640	0.973821	0.733691	0.223*	
H24C	0.068855	0.898752	0.765799	0.223*	

### Atomic displacement parameters (Å^2^)

**Table:**

	*U*^11^	*U*^22^	*U*^33^	*U*^12^	*U*^13^	*U*^23^
Cu1	0.0357 (4)	0.0410 (5)	0.0215 (4)	0.0001 (4)	0.0133 (3)	0.0025 (4)
N1	0.069 (4)	0.044 (4)	0.030 (3)	−0.013 (3)	0.027 (3)	−0.006 (3)
N2	0.051 (4)	0.086 (5)	0.050 (4)	−0.012 (4)	0.007 (3)	0.002 (4)
O1	0.050 (3)	0.054 (3)	0.028 (3)	−0.010 (2)	0.012 (2)	0.000 (2)
O2	0.047 (3)	0.056 (3)	0.031 (3)	−0.012 (2)	0.013 (2)	0.005 (2)
O5	0.045 (3)	0.054 (3)	0.028 (3)	0.009 (2)	0.016 (2)	0.003 (2)
O6	0.046 (3)	0.055 (3)	0.032 (3)	0.011 (2)	0.017 (2)	0.002 (2)
O7	0.155 (7)	0.145 (7)	0.075 (5)	0.000 (6)	0.077 (5)	0.017 (5)
O8	0.067 (4)	0.069 (4)	0.075 (4)	−0.006 (3)	0.038 (3)	0.003 (3)
O9	0.041 (2)	0.059 (3)	0.026 (2)	0.001 (2)	0.015 (2)	0.005 (2)
O10	0.072 (4)	0.133 (7)	0.115 (6)	−0.044 (5)	0.013 (4)	0.008 (5)
C1	0.031 (3)	0.039 (4)	0.040 (4)	−0.001 (3)	0.012 (3)	−0.002 (3)
C2	0.036 (3)	0.037 (4)	0.034 (4)	−0.007 (3)	0.016 (3)	0.000 (3)
C3	0.045 (4)	0.056 (5)	0.041 (5)	−0.007 (4)	0.014 (3)	0.005 (4)
C4	0.049 (4)	0.064 (5)	0.044 (5)	−0.012 (4)	0.008 (4)	−0.005 (4)
C5	0.045 (4)	0.064 (6)	0.064 (6)	−0.016 (4)	0.024 (4)	−0.013 (4)
C6	0.055 (5)	0.054 (5)	0.053 (5)	−0.007 (4)	0.020 (4)	0.004 (4)
C7	0.046 (4)	0.049 (5)	0.047 (5)	−0.009 (4)	0.011 (3)	−0.002 (4)
C8	0.058 (8)	0.038 (7)	0.050 (9)	−0.008 (5)	0.019 (7)	0.006 (6)
O3	0.088 (6)	0.070 (6)	0.048 (5)	−0.007 (4)	0.040 (4)	−0.008 (4)
O4	0.072 (7)	0.065 (6)	0.036 (6)	0.017 (5)	0.001 (5)	−0.004 (5)
C9	0.101 (13)	0.122 (11)	0.049 (9)	0.060 (11)	−0.008 (7)	−0.016 (8)
C8A	0.08 (4)	0.03 (2)	0.05 (3)	0.02 (2)	0.03 (3)	0.00 (2)
O3A	0.043 (15)	0.10 (2)	0.045 (17)	0.011 (12)	0.013 (12)	−0.008 (14)
O4A	0.070 (9)	0.064 (7)	0.035 (7)	0.010 (7)	0.000 (6)	−0.001 (6)
C9A	0.101 (13)	0.122 (11)	0.048 (9)	0.060 (11)	−0.007 (7)	−0.016 (8)
C10	0.028 (3)	0.048 (4)	0.031 (4)	−0.001 (3)	0.008 (3)	−0.003 (3)
C11	0.044 (4)	0.054 (5)	0.034 (4)	0.009 (3)	0.012 (3)	−0.004 (3)
C12	0.064 (5)	0.071 (6)	0.064 (6)	0.008 (5)	0.030 (4)	0.008 (5)
C13	0.097 (7)	0.059 (6)	0.103 (8)	0.028 (6)	0.034 (7)	0.008 (6)
C14	0.101 (8)	0.098 (9)	0.092 (8)	0.050 (7)	0.040 (7)	0.001 (7)
C15	0.073 (6)	0.104 (8)	0.073 (7)	0.032 (6)	0.043 (5)	0.008 (6)
C16	0.046 (4)	0.073 (6)	0.040 (4)	0.019 (4)	0.018 (4)	−0.001 (4)
C17	0.044 (4)	0.105 (8)	0.052 (5)	0.012 (5)	0.028 (4)	0.007 (5)
C18	0.080 (7)	0.088 (8)	0.139 (10)	0.001 (6)	0.067 (7)	0.012 (7)
C19	0.047 (4)	0.053 (5)	0.033 (4)	0.000 (3)	0.016 (3)	−0.004 (3)
C20	0.096 (6)	0.086 (7)	0.040 (5)	0.016 (5)	0.030 (5)	0.025 (5)
C21	0.103 (7)	0.079 (6)	0.077 (6)	−0.021 (5)	0.065 (6)	−0.008 (5)
C22	0.091 (8)	0.075 (7)	0.085 (8)	−0.003 (6)	0.000 (7)	−0.004 (6)
C23	0.066 (6)	0.158 (11)	0.100 (8)	−0.007 (7)	0.039 (6)	−0.022 (8)
C24	0.097 (8)	0.29 (2)	0.067 (8)	−0.016 (10)	0.040 (7)	−0.015 (10)

### Geometric parameters (Å, º)

**Table:**

Cu1—Cu1^i^	2.6329 (14)	C9—H9B	0.9600
Cu1—O1	1.943 (4)	C9—H9C	0.9600
Cu1—O2^i^	1.934 (4)	C8A—O3A	1.17 (5)
Cu1—O5	1.968 (4)	C8A—O4A	1.28 (6)
Cu1—O6^i^	1.953 (4)	O4A—C9A	1.50 (5)
Cu1—O9	2.132 (4)	C9A—H9A1	0.9600
N1—C19	1.297 (8)	C9A—H9A2	0.9600
N1—C20	1.442 (10)	C9A—H9A3	0.9600
N1—C21	1.448 (9)	C10—C11	1.488 (9)
N2—C22	1.306 (10)	C11—C12	1.363 (10)
N2—C24	1.401 (11)	C11—C16	1.395 (9)
N2—C23	1.421 (10)	C12—C13	1.381 (11)
O1—C1	1.247 (7)	C12—H12	0.9300
O2—C1	1.266 (7)	C13—C14	1.367 (13)
O5—C10	1.251 (7)	C13—H13	0.9300
O6—C10	1.245 (7)	C14—C15	1.335 (13)
O7—C17	1.176 (9)	C14—H14	0.9300
O8—C17	1.313 (10)	C15—C16	1.366 (11)
O8—C18	1.420 (10)	C15—H15	0.9300
O9—C19	1.234 (7)	C16—C17	1.480 (12)
O10—C22	1.169 (11)	C18—H18A	0.9600
C1—C2	1.485 (9)	C18—H18B	0.9600
C2—C3	1.359 (9)	C18—H18C	0.9600
C2—C7	1.393 (9)	C19—H19	0.9300
C3—C4	1.374 (9)	C20—H20A	0.9600
C3—H3	0.9300	C20—H20B	0.9600
C4—C5	1.357 (10)	C20—H20C	0.9600
C4—H4	0.9300	C21—H21A	0.9600
C5—C6	1.370 (10)	C21—H21B	0.9600
C5—H5	0.9300	C21—H21C	0.9600
C6—C7	1.367 (9)	C22—H22	0.9300
C6—H6	0.9300	C23—H23A	0.9600
C7—C8	1.513 (16)	C23—H23B	0.9600
C7—C8A	1.55 (5)	C23—H23C	0.9600
C8—O3	1.179 (15)	C24—H24A	0.9600
C8—O4	1.349 (19)	C24—H24B	0.9600
O4—C9	1.442 (16)	C24—H24C	0.9600
C9—H9A	0.9600		
			
O2^i^—Cu1—O1	168.03 (18)	O4A—C9A—H9A2	109.5
O2^i^—Cu1—O6^i^	90.25 (19)	H9A1—C9A—H9A2	109.5
O1—Cu1—O6^i^	89.05 (19)	O4A—C9A—H9A3	109.5
O2^i^—Cu1—O5	88.7 (2)	H9A1—C9A—H9A3	109.5
O1—Cu1—O5	89.48 (19)	H9A2—C9A—H9A3	109.5
O6^i^—Cu1—O5	168.06 (18)	O6—C10—O5	126.1 (6)
O2^i^—Cu1—O9	99.14 (18)	O6—C10—C11	116.6 (6)
O1—Cu1—O9	92.82 (18)	O5—C10—C11	117.1 (6)
O6^i^—Cu1—O9	96.36 (17)	C12—C11—C16	119.2 (7)
O5—Cu1—O9	95.55 (17)	C12—C11—C10	119.6 (6)
O2^i^—Cu1—Cu1^i^	85.81 (13)	C16—C11—C10	121.2 (7)
O1—Cu1—Cu1^i^	82.24 (13)	C11—C12—C13	121.2 (8)
O6^i^—Cu1—Cu1^i^	83.61 (13)	C11—C12—H12	119.4
O5—Cu1—Cu1^i^	84.46 (13)	C13—C12—H12	119.4
O9—Cu1—Cu1^i^	175.05 (13)	C14—C13—C12	118.1 (9)
C19—N1—C20	121.4 (6)	C14—C13—H13	120.9
C19—N1—C21	120.8 (7)	C12—C13—H13	120.9
C20—N1—C21	117.6 (6)	C15—C14—C13	121.4 (9)
C22—N2—C24	120.9 (9)	C15—C14—H14	119.3
C22—N2—C23	122.1 (9)	C13—C14—H14	119.3
C24—N2—C23	117.1 (8)	C14—C15—C16	121.5 (8)
C1—O1—Cu1	125.6 (4)	C14—C15—H15	119.3
C1—O2—Cu1^i^	121.4 (4)	C16—C15—H15	119.3
C10—O5—Cu1	122.0 (4)	C15—C16—C11	118.6 (8)
C10—O6—Cu1^i^	123.9 (4)	C15—C16—C17	118.1 (7)
C17—O8—C18	117.6 (7)	C11—C16—C17	123.1 (7)
C19—O9—Cu1	120.4 (4)	O7—C17—O8	124.0 (10)
O1—C1—O2	125.0 (6)	O7—C17—C16	124.6 (10)
O1—C1—C2	117.1 (6)	O8—C17—C16	111.3 (7)
O2—C1—C2	118.0 (6)	O8—C18—H18A	109.5
C3—C2—C7	118.5 (6)	O8—C18—H18B	109.5
C3—C2—C1	120.5 (6)	H18A—C18—H18B	109.5
C7—C2—C1	120.9 (6)	O8—C18—H18C	109.5
C2—C3—C4	120.6 (7)	H18A—C18—H18C	109.5
C2—C3—H3	119.7	H18B—C18—H18C	109.5
C4—C3—H3	119.7	O9—C19—N1	124.1 (7)
C5—C4—C3	120.6 (7)	O9—C19—H19	118.0
C5—C4—H4	119.7	N1—C19—H19	118.0
C3—C4—H4	119.7	N1—C20—H20A	109.5
C4—C5—C6	119.8 (7)	N1—C20—H20B	109.5
C4—C5—H5	120.1	H20A—C20—H20B	109.5
C6—C5—H5	120.1	N1—C20—H20C	109.5
C7—C6—C5	119.8 (7)	H20A—C20—H20C	109.5
C7—C6—H6	120.1	H20B—C20—H20C	109.5
C5—C6—H6	120.1	N1—C21—H21A	109.5
C6—C7—C2	120.6 (7)	N1—C21—H21B	109.5
C6—C7—C8	115.1 (7)	H21A—C21—H21B	109.5
C2—C7—C8	123.7 (7)	N1—C21—H21C	109.5
C6—C7—C8A	119.2 (17)	H21A—C21—H21C	109.5
C2—C7—C8A	113.0 (18)	H21B—C21—H21C	109.5
O3—C8—O4	123.5 (13)	O10—C22—N2	129.6 (11)
O3—C8—C7	128.7 (13)	O10—C22—H22	115.2
O4—C8—C7	107.7 (12)	N2—C22—H22	115.2
C8—O4—C9	116.9 (13)	N2—C23—H23A	109.5
O4—C9—H9A	109.5	N2—C23—H23B	109.5
O4—C9—H9B	109.5	H23A—C23—H23B	109.5
H9A—C9—H9B	109.5	N2—C23—H23C	109.5
O4—C9—H9C	109.5	H23A—C23—H23C	109.5
H9A—C9—H9C	109.5	H23B—C23—H23C	109.5
H9B—C9—H9C	109.5	N2—C24—H24A	109.5
O3A—C8A—O4A	126 (5)	N2—C24—H24B	109.5
O3A—C8A—C7	131 (5)	H24A—C24—H24B	109.5
O4A—C8A—C7	102 (4)	N2—C24—H24C	109.5
C8A—O4A—C9A	113 (4)	H24A—C24—H24C	109.5
O4A—C9A—H9A1	109.5	H24B—C24—H24C	109.5
			
Cu1—O1—C1—O2	−2.2 (9)	O3A—C8A—O4A—C9A	5 (6)
Cu1—O1—C1—C2	179.2 (4)	C7—C8A—O4A—C9A	−180 (3)
Cu1^i^—O2—C1—O1	1.3 (9)	Cu1^i^—O6—C10—O5	−1.1 (9)
Cu1^i^—O2—C1—C2	179.8 (4)	Cu1^i^—O6—C10—C11	172.8 (4)
O1—C1—C2—C3	159.7 (6)	Cu1—O5—C10—O6	0.9 (9)
O2—C1—C2—C3	−18.9 (9)	Cu1—O5—C10—C11	−173.0 (4)
O1—C1—C2—C7	−16.9 (9)	O6—C10—C11—C12	−41.0 (9)
O2—C1—C2—C7	164.5 (6)	O5—C10—C11—C12	133.5 (7)
C7—C2—C3—C4	0.2 (10)	O6—C10—C11—C16	141.9 (7)
C1—C2—C3—C4	−176.5 (6)	O5—C10—C11—C16	−43.6 (9)
C2—C3—C4—C5	−0.3 (11)	C16—C11—C12—C13	1.3 (12)
C3—C4—C5—C6	0.2 (11)	C10—C11—C12—C13	−175.9 (8)
C4—C5—C6—C7	−0.1 (11)	C11—C12—C13—C14	−0.9 (14)
C5—C6—C7—C2	0.0 (11)	C12—C13—C14—C15	1.1 (17)
C5—C6—C7—C8	172.2 (9)	C13—C14—C15—C16	−1.9 (17)
C5—C6—C7—C8A	−148 (2)	C14—C15—C16—C11	2.2 (14)
C3—C2—C7—C6	−0.1 (10)	C14—C15—C16—C17	−173.3 (9)
C1—C2—C7—C6	176.6 (6)	C12—C11—C16—C15	−1.9 (11)
C3—C2—C7—C8	−171.6 (9)	C10—C11—C16—C15	175.2 (7)
C1—C2—C7—C8	5.1 (12)	C12—C11—C16—C17	173.4 (8)
C3—C2—C7—C8A	150.1 (19)	C10—C11—C16—C17	−9.5 (11)
C1—C2—C7—C8A	−33 (2)	C18—O8—C17—O7	−0.5 (12)
C6—C7—C8—O3	−74.5 (14)	C18—O8—C17—C16	−177.6 (6)
C2—C7—C8—O3	97.4 (13)	C15—C16—C17—O7	−51.0 (12)
C6—C7—C8—O4	101.3 (10)	C11—C16—C17—O7	133.7 (9)
C2—C7—C8—O4	−86.8 (12)	C15—C16—C17—O8	126.0 (8)
O3—C8—O4—C9	−2 (2)	C11—C16—C17—O8	−49.3 (10)
C7—C8—O4—C9	−178.1 (12)	Cu1—O9—C19—N1	171.9 (5)
C6—C7—C8A—O3A	106 (4)	C20—N1—C19—O9	−2.4 (11)
C2—C7—C8A—O3A	−45 (5)	C21—N1—C19—O9	−177.0 (7)
C6—C7—C8A—O4A	−70 (3)	C24—N2—C22—O10	−2.6 (18)
C2—C7—C8A—O4A	139 (2)	C23—N2—C22—O10	176.5 (12)

Symmetry code: (i) −*x*+1, −*y*+1, −*z*+1.

### Hydrogen-bond geometry (Å, º)

**Table:**

*D*—H···*A*	*D*—H	H···*A*	*D*···*A*	*D*—H···*A*
C12—H12···O3*A*^i^	0.93	2.50	3.29 (3)	143
C23—H23*A*···O6^ii^	0.96	2.70	3.538 (11)	145
C3—H3···O10^iii^	0.93	2.63	3.284 (10)	128
C6—H6···O10^iv^	0.93	2.69	3.355 (11)	129

Symmetry codes: (i) −*x*+1, −*y*+1, −*z*+1; (ii) *x*, −*y*+3/2, *z*+1/2; (iii) −*x*, *y*−1/2, −*z*+1/2; (iv) −*x*, −*y*+1, −*z*+1.
